# A hybrid parallel convolutional spiking neural network for enhanced skin cancer detection

**DOI:** 10.1038/s41598-025-85627-6

**Published:** 2025-04-01

**Authors:** K. Anup Kumar, C. Vanmathi

**Affiliations:** School of Computer Science Engineering and Information Systems, Vellore Institute of Technology, Vellore, Tamilnadu India

**Keywords:** Parallel convolutional neural network, Deep spiking neural network, SegNet, Deep joint segmentation, RV coefficient, Skin cancer, Cancer

## Abstract

The most widespread kind of cancer, affecting millions of lives is skin cancer. When the condition of illness worsens, the chance of survival is reduced, and thus detection of skin cancer is extremely difficult. Hence, this paper introduces a new model, known as Parallel Convolutional Spiking Neural Network (PCSN-Net) for detecting skin cancer. Initially, the input skin cancer image is pre-processed by employing Medav filter to eradicate the noise in image. Next, affected region is segmented by utilizing DeepSegNet, which is formed by integrating SegNet and Deep joint segmentation, where RV coefficient is used to fuse the outputs. Here, the segmented image is then augmented by including process, such as geometric transformation, colorspace transformation, mixing images Pixel averaging (mixup), and overlaying crops (CutMix). Then textural, statistical, Discrete Wavelet Transform (DWT) based Local Direction Pattern (LDP) with entropy, and Local Normal Derivative Pattern (LNDP) features are mined. Finally, skin cancer detection is executed using PCSN-Net, which is formed by fusing Parallel Convolutional Neural Network (PCNN) and Deep Spiking Neural Network (DSNN). In this work, the suggested PCSN-Net system shows high accuracy and reliability in identifying skin cancer. The experimental findings suggest that PCSN-Net has an accuracy of 95.7%, a sensitivity of 94.7%, and a specificity of 92.6%. These parameters demonstrate the model’s capacity to discriminate among malignant and benign skin lesions properly. Furthermore, the system has a false positive rate (FPR) of 10.7% and a positive predictive value (PPV) of 90.8%, demonstrating its capacity to reduce wrong diagnosis while prioritizing true positive instances. PCSN-Net outperforms various complex algorithms, including EfficientNet, DenseNet, and Inception-ResNet-V2, despite preserving effective training and inference times. The results obtained show the feasibility of the model for real-time clinical use, strengthening its capacity for quick and accurate skin cancer detection.

## Introduction

Skin cancer is increasing development of skin cells because of inherited or alteration irregularity of damaged Deoxyribonucleic Acid (DNA). Generally. Skin cancer is fatal of all other types of cancerous cells. According to the World Health Organization (WHO), about 1/3 of total cancer cases are determined globally are skin cancer^1^. Generally, the malignant stage of skin cancer is caused due to pigmented cells termed melanocytes^2^. Skin cancer is commonly found in different colors, like royal purple, azure, and rosy pink, or it can also be colorless. The rapid spreading ability of skin cancer makes it more fatal and dangerous than other cancers^3^. Skin cancer mainly occurs due to several hereditary and environmental factors, namely the exposure of harmful Ultraviolet (UV) radiation from the sun, particularly short wavelength of UV-B and long wavelength of UV-A. These radiations from the sun lead to an increase in the growth of the melanocytes, which are the pigment generating cells of human skin. The most commonly reported skin cancerous cells are Squamous Cell Carcinoma (SCC), Basal Cell Carcinoma (BCC), and malignant melanoma^4^. The SCC and BCC are non-melanocytic cancers among all these different skin cancers, which are also considered innocuous. Similarly, malignant melanoma is considered the most life-threatening skin cancer with increasing death cases^1^.

Malignant melanoma is grave kind of skin cancer and the exact underlying reason of malignant melanoma is unknown. However, various factors, like environmental contact, ultraviolet radiation, genetic factors, and so on are considered to increase its occurrence^4^. Based on the American Cancer Society’s (ACS) 2017 report, it is estimated that a person dies due to melanoma every 54–60 min. The annual report of ACS 2019 determined that there are 7230 deaths from melanoma among 96,480 new melanoma cases globally. Skin cancer can be cured effectively if recognized and detected at the initial stages. If the diagnosis is in situ, then the melanoma survival rate is about 95%. Likewise, the survival rate reduces to 15%, if the disease is diagnosed at primary stage. Skin cancer decreases the mortality rate of the person to less than 20%, which may also metastate to liver or lung cancer if the tumor is not treated at the initial stages^1^. In general, skin cancer spreads throughout its surroundings under severe cases. Meanwhile, the chances of survival rate are high, if the disease is detected and treatment is provided at initial stages^5^. The melanoma moles are found in different shapes as well as colors based on disease severity level, like brown, black, red, pink, and so on. The expected melanoma infection is determined by measuring the diameter of the abnormal color mole of more than 6 mm by the dermatologist. At first, the diameter, abnormal skin color, size, shape, and behavior of the mole are visually examined by a dermatologist^6^.

In recent years, the detection of skin cancer at the initial stage has been a crucial subject of research^5^. The early diagnosis of skin cancer is a difficult task due to the advent of diverse kinds of skin lesions, mainly carcinoma and melanoma^7^. The mammography images are screened and validated by clinical specimens to typically detect skin cancer by ensuring better death rates as well as prognosis^8^. Over past decades, dermoscopy images which is a non-trauma skin imaging approaches are most commonly exploited for the diagnosis of skin cancer. As compared with other auxiliary observations, the accuracy of dermoscopy images is higher to identify skin cancer from dermoscopy images^1^. However, diagnostic accuracy is based on the professional skills and experience of dermatologists^9^. Moreover, various noninvasive techniques are developed to avoid unnecessary biopsy to diagnose skin cancer^7^. Nowadays, Computer-Aided Design (CAD) techniques that are more consistent, reliable, and quick in diagnosing different disorders are used to diagnose skin cancer at the initial stages. The CAD techniques diagnose tumor disease more accurately in a cost-effective manner^8^. Meanwhile, the detection is performed manually, which also takes more time to perform the detection task. At present, machine learning and deep learning techniques are used for the detection task due to the increasing advancement of artificial intelligence. The machine learning models precisely detect skin cancer and classify using different techniques, like Support Vector Machines (SVM), Naïve Bayes (NB), Decision Trees (DT) classifiers, and so on. Likewise, the deep learning technique, Convolutional Neural Networks (CNN) has gained the attention due to their capability to automatically extract features. The CNN helps to identify cancerous cells more effectively and rapidly^10^. The recent breakthrough of deep learning techniques surpasses the detection accuracy attained by expert-levels not only in skin cancer but also in other pathologies. For example, CNN is well capable of matching the classification accuracy of dermatologist-level from the large number of skin lesion databases^11^. The aim is to devise a new approach termed PCSN-Net for skin cancer detection.

The contribution of this work is given below,

- The skin cancer image is pre-processed by employing Medav filter to eradicate the noise.
- The cancer region is segmented by utilizing DeepSegNet, which is formed by integrating SegNet and Deep joint segmentation, where RV coefficient is used to fuse the outputs.
- Later, segmented image is augmented by including the process, namely CutMix, geometric transformation, mixup, and colorspace transformation.
- Then the features, such as textural, statistical, LNDP, DWT based LDP with entropy are mined.
- At last, skin cancer detection is performed by employing PCSN-Net, which is formed by fusing PCNN and DSNN.

The rest of the process are organized as: Section 2 displays challenges faced by other systems; Section 3 illustrates description of PCSN-Net for detecting skin cancer, Section 4 shows discussion of experimental outcomes, and Section 5 portrays conclusion of this paper.

## Motivation

Over past decades, several CAD techniques were introduced to accurately detect skin cancer from skin cancer images. Meanwhile, the automatic segmentation and localization of skin lesions at the initial phases due to the high level of color similarity between melanoma-affected regions posed a great challenge. Also, these techniques encountered overfitting issues and recorded high computational costs while detecting skin cancer. Hence, it motivates to develop deep-learning model to accurately identify skin cancer from skin images. The motivation behind using Spiking Neural Networks (SNNs) in this study lies in their energy-efficient, biologically inspired processing, which is advantageous for resource-constrained environments like portable diagnostic devices in remote healthcare. SNNs operate with sparse, event-driven computation, resulting in lower power consumption compared to traditional neural networks. Additionally, SNNs excel at handling temporal data and asynchronous processing, making them suitable for real-time diagnostic applications that require immediate feedback, such as telemedicine consultations. Their brain-like structure allows for more biologically plausible computations, potentially enhancing model interpretability—critical in medical fields where trust and transparency are essential. Furthermore, SNNs have shown robustness in handling noisy or sparse data, making them highly effective for analyzing medical images that may vary in quality. These unique advantages justify the use of SNNs, providing not only accurate skin cancer detection but also enhanced efficiency, interpretability, and applicability in real-world healthcare settings.

### Literature survey

Rehan Ashraf et al.^3^ designed a transfer learning model for identifying skin cancer. In this model, the melanoma was discriminated using intelligent Region of Interest (RoI). This model effectively prevented unbalancing problems and overfitting issues while accurately detecting melanoma. Meanwhile, it failed to utilize a real-time database for detecting skin cancer from the most discriminative features. Ni Zhang et al.^4^ established a CNN model for early identification of skin cancer. Here, an improved Whale optimization algorithm (WOA) technique was utilized to select optimal CNN parameters. This model was highly significant in diagnosing skin cancer under minimum error from skin images and was more suitable for diagnosing skin cancer under various environmental conditions. However, this approach failed to attain high accuracy while diagnosing skin cancer. Lisheng Wei et al.^9^ developed an ensemble lightweight deep learning to automatically detect skin cancer from dermoscopic images. This technique utilized a small quantity of parameters to accurately detect skin cancer. Meanwhile, it failed to meet the expectations by adjusting the hyperparameters, which was unbalanced during training. Marriam Nawaz et al.^6^ introduced a deep learning approach to detect skin cancer by utilizing dermoscopic images. This approach was highly robust for the recognition and segmentation of skin lesion regions, but it recorded high computational complications during the detection of fatal skin cancer. Altaheri et al.^12^ developed the Dynamic Attention Temporal Convolutional Network (D-ATCNet) to improve EEG-based MI decoding. D-ATCNet uses dynamic convolution and multilayer attention methods to increase precision in classification with fewer parameters. It has two primary elements: a dynamic convolution block with multilevel attention for encoding low-level MI-EEG information, and a temporal convolution block that uses shifting window self-attention to obtain high-level spatial properties. Conze et al.^13^ present a thorough analysis of advances in segmenting medical images employing deep learning. They examine the transition of U-Net architectures to further advanced models that include conditional generative adversarial networks (cGANs), cascaded networks, and Transformers. They also look at developing methods including contrastive learning, knowledge distillation, and semi-supervised learning, emphasising their capacity to improve segmentation accuracy and generalization. They also address concerns such as as limited data and prior knowledge integration, suggesting methods such as cross-modality learning and federated learning to overcome these obstacles. Sun et al.^14^ developed a memristive fully interconnected neural network (MFNN) specifically designed for medical picture encryption. They created a flux-controlled hyperbolic memristor system that had complex nonlinear properties, that was subsequently incorporated into a Hopfield neural network to construct the MFNN. The network itself showed sophisticated nonlinear practices, which were verified using computational simulations and circuit designs. They suggested an encryption technique that uses chaotic sequences for bit-level permutation, and then diffusion using a central diffusion algorithm. Performance studies revealed strong security, including a data entropy of 7.99 and low correlation coefficients, demonstrating the system’s resistance to statistical threats.

Singh et al.^1^ designed You Look Only Once-Deep CNN (YOLO-DCNN) for identifying skin cancer from dermoscopic and digital lesion images. This approach effectively captured the local and global features under less complexity. Meanwhile, it failed to determine the nature of lesions among melanoma patients. Tahir et al.^10^ developed a Deep Learning-based Skin Cancer Classification Network (DSCC_Net) technique to accurately diagnose skin cancer. This approach significantly handled the minority class issues in the database and reduced the complexity by reducing the total number of trainable parameters. However, this model failed to attain favorable results during classification by incorporating federated learning and blockchain for skin infection detection and skin cancer classification. Imran et al.^15^ established an ensemble model for the detection of skin cancer by utilizing dermoscopic images. This model recorded less training time for detecting skin cancer at early stages, but it failed to consider reinforcement learning-based models to increase the detection accuracy of skin cancer. Lu and Zadeh et al.^7^ introduced XceptionNet for early diagnosis of skin cancer. This mode recorded low overfitting issues and converged faster during the determination of non-linear features from the database while detecting skin cancer. Meanwhile, this model was expensive due to the training of complex data for the diagnosis of skin cancer. Li et al.^16^ created a deep recurrent-convolutional neural network (DRCNN) for EEG-based intention recognition by combining convolutional and recurrent levels to collect both temporal and spatial aspects of EEG data. They utilized Gradient-weighted Class Activation Mapping (Grad-CAM) to identify and choose those with the most relevant EEG methods, which improved model understanding and efficiency. When tested on the EEGMMIDB dataset, the DRCNN attained a detection accuracy of 97.76%, outperforming numerous modern techniques. Zhang et al.^17^ developed a hybrid neural network system to categorize four-class motor imagery (MI) tasks based on electroencephalogram (EEG) inputs. The method blends a one-versus-rest filter bank similar spatial structure (OVR-FBCSP) for feature extraction with a hybrid deep neural network that incorporates convolutional neural networks (CNNs) and long short-term memory (LSTM) networks. This method captures both the spatial and temporal characteristics of MI-EEG data. Sun et al.^18^ developed a memristor-based neural network that mimics behavioral conditioning behaviors, with a focus on preventing and competitive impacts. The framework includes memristive synapses to replicate synaptic plasticity, allowing for the simulation of complicated process of learning found in biological structures. By combining such effects, the system can distinguish between stimuli according to their association intensity, thereby replicating the selective learning found in wild creatures.

### Challenges

The drawbacks comforted by baseline schemes utilized to detect skin cancer are enumerated below,

- The transfer learning model employed in^3^ enhanced the performance by reducing overfitting and imbalance issues during skin cancer detection, but this approach was performed only on the data that comprised skin cancer areas.
- The optimized CNN technique used in^4^ effectively minimized error while diagnosing skin cancer. Meanwhile, this approach was not successful in utilizing hybrid deep learning techniques for the detection of skin cancer.
- The ensemble lightweight deep learning model used in^9^ failed to include devising highly efficient discrimination networks and failed to assess the method on several datasets.
- The deep learning approach used in^6^ was highly efficient in detecting and segmenting skin lesions in earlier stages, though it failed to evaluate the method on larger datasets during the detection of skin cancer.
- The baseline techniques used for skin cancer detection significantly decreased human error, and enhanced the speed of diagnosis process. Despite these benefits, these techniques suffered from poor discrimination issues among different features used for detection of skin cancer.

## Proposed PCSN-Net for skin cancer detection

**Fig. 1:**
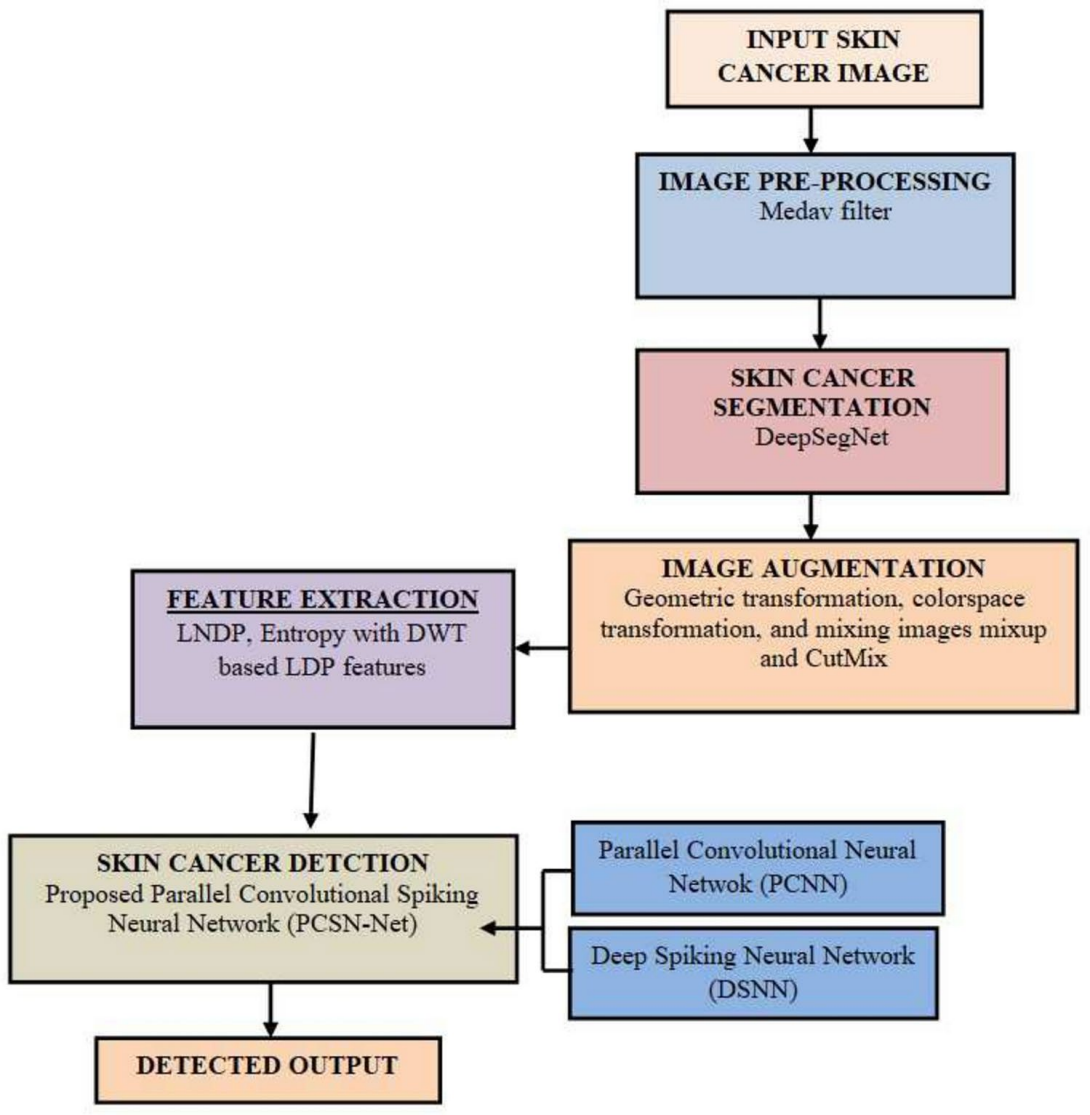
Block diagram of the proposed PCSN-Net for skin cancer detection.

A novel DL technique called PCSN-Net is presented for skin cancer detection. For that, firstly, the input skin cancer image acquired from the database is initially allowed for image pre-processing using the Medav filter^19^ for denoising the naturally available noise present in the input image. Then, preprocessed skin image is given to DeepSegNet for skin cancer segmentation. The DeepSegNet is designed by using SegNet^20,21^ and Deep joint segmentation^22^ using RV coefficient^23^. The resultant segmented skin cancer images are further sent to data augmentation process, where the augmentation processes like mixup, geometric transformation, CutMix, and colorspace transformation are utilized for increasing the number of training samples. Later, the different feature extractors are utilized to extract texture features^24^, such as contrast, correlation, energy, and homogeneity, and statistical features^24^, like mean, variance, and skewness, as well as LNDP^25^, Entropy^26^ with DWT^27^ based LDP features^28^. Finally, skin cancer detection is executed by PCSN-Net. The PCSN-Net model is designed by the incorporation of PCNN^29^ and DSNN^30,31^. Figure 1 shows block diagram of PCSN-Net for skin cancer detection.

### Training methodology of PCSN-Net

The PCSN-Net model for skin cancer detection is trained using an extensive approach that aims at improving the detection rate while efficiently processing complicated picture information. At first, the input photos are pre-processed with a Medav filter, which combines median and mean filters to eliminate noise while preserving significant information. This pre-processed picture is then segmented with DeepSegNet, a network that uses SegNet and Deep Joint Segmentation to reliably identify damaged regions. The RV coefficient is used to combine SegNet and Deep Joint Segmentation results, improving segmentation precision. To increase model resilience, data augmentation techniques are used to create larger numbers of training data sets. These consist of geometric transformations, Mixup, CutMix, and colorspace alterations (such as brightness and hue shifts). These augmentations give a more diverse set of training pictures that more accurately reflect real-world variability, lowering the risk of overfitting. Feature extraction aims to capture important image properties. Textural data such as contrast, correlation, energy, and homogeneity are extracted, as well as statistical features (mean, variance, skewness) and wavelet-based features (DWT with Local Direction Pattern and entropy). These characteristics combine to enhance the dataset with discriminative information required for accurate classification. The system’s architecture combines a Parallel Convolutional Neural Network (PCNN) and a Deep Spiking Neural Network (DSNN). The PCNN analyses spatial data with convolutional layers, whereas the DSNN’s spiking neurons capture temporal patterns with energy-efficient processing. To improve the accuracy of detection, critical parameters such as learning rate, batch size, and number of layers are tuned during training. The model that results has excellent accuracy and specificity for differentiating between malignant and benign skin lesions, and it performs well in real-world tests. Table 1 shows Parameter Specifications for PCSN-Net Model Components.

**Table 1:** The parameter specifications for PCSN-Net model components.

Layer/component	Parameter	Value/description
Image pre-processing	Filter type	Medav filter
	Mean filter size	$3 \times 3$
	Median filter size	$3 \times 3$
Segmentation	Model	DeepSegNet
	Encoder depth	3 layers
	Decoder depth	3 layers
	Fusion coefficient	RV coefficient
Image augmentation	Techniques	Mixup, CutMix, geometric transformation, colorspace transformation
	Rotation angle	$0^{\circ }$ to $90^{\circ }$
	Brightness range	$\pm 0.2$
Feature extraction	Texture features	Contrast, correlation, energy, homogeneity
	Statistical features	Mean, variance, skewness
PCNN model	Convolutional layers	Number of layers: 4
	Filter size	$3 \times 3$
	Strides	1
	Activation function	ReLU
	Pooling	Max pooling (size: $2 \times 2$)
PCSN-net layer	Fusion method	Regression via fractional calculus
	Statistical and textural features	7 features
DSNN model	Neuron firing threshold	0.5
	Spike count learning rule	Spike vector quantization
	Synaptic weights initialization	Xavier initialization
	Spiking activation	Spiking ReLU

### Image acquisition

Skin cancer image from database^32^ is employed for detecting skin cancer, and it is expressed as,

1 $$\begin{aligned} O=\{ O_1, O_2,..., O_f,...,O_a \} \end{aligned}$$

here, $O_f$ symbolizes the $f^{th}$ image, which is used for detecting skin cancer, and overall amount of image is represented as *a*.

### Image pre-processing

Pre-processing is employed to advance the image, which are important for the subsequent processes and also it aims in enhancing quality of image by removing unwanted alterations. Moreover, it enhanced model inference and lessened the quantity of time required during training. The input for pre-processing is considered as $O_f$, and the process is performed by Medav Filter^19^. To change the level of mask operations in terms of noise density, Medav Filter^19^ is created by combining mean filter and median filter. Here, mean filter is utilized to identify noise contained in image, however it is incapable to eradicate highly tailored noise, while median filter successfully reduces noise but its complication is not desired. Thus, the complexity is decreased and the performance is enhanced by integrating these two methods. In addition to this, pixel value is related to average value $H_{avg}^\prime$ after the calculation of mask input’s average value. If the value is high, median value $H_{med}^\prime$ is utilized to substitute the pixel or else the original value is used, and *M* characterizes the pre-processed image.

### Cancer region segmentation

Image segmentation is more effective, and significant, and it is widely used in several clinical image processing tasks, namely lesion tissue detection, quantitative assessment, tumor radiotherapies, therapy estimation, and so on. The segmentation of cancer region from an image is employed to identify the skin cancer appropriately. Moreover, SegNet^20,21^ and DeepJoint segmentation^22^ is used to segment the cancer region, further RV coefficient is utilized for fusing the outcomes of these approaches. Moreover, pre-processed image is taken as input for segmentation, and a detailed explanation is given below.

#### Architecture of SegNet

**Fig. 2:**
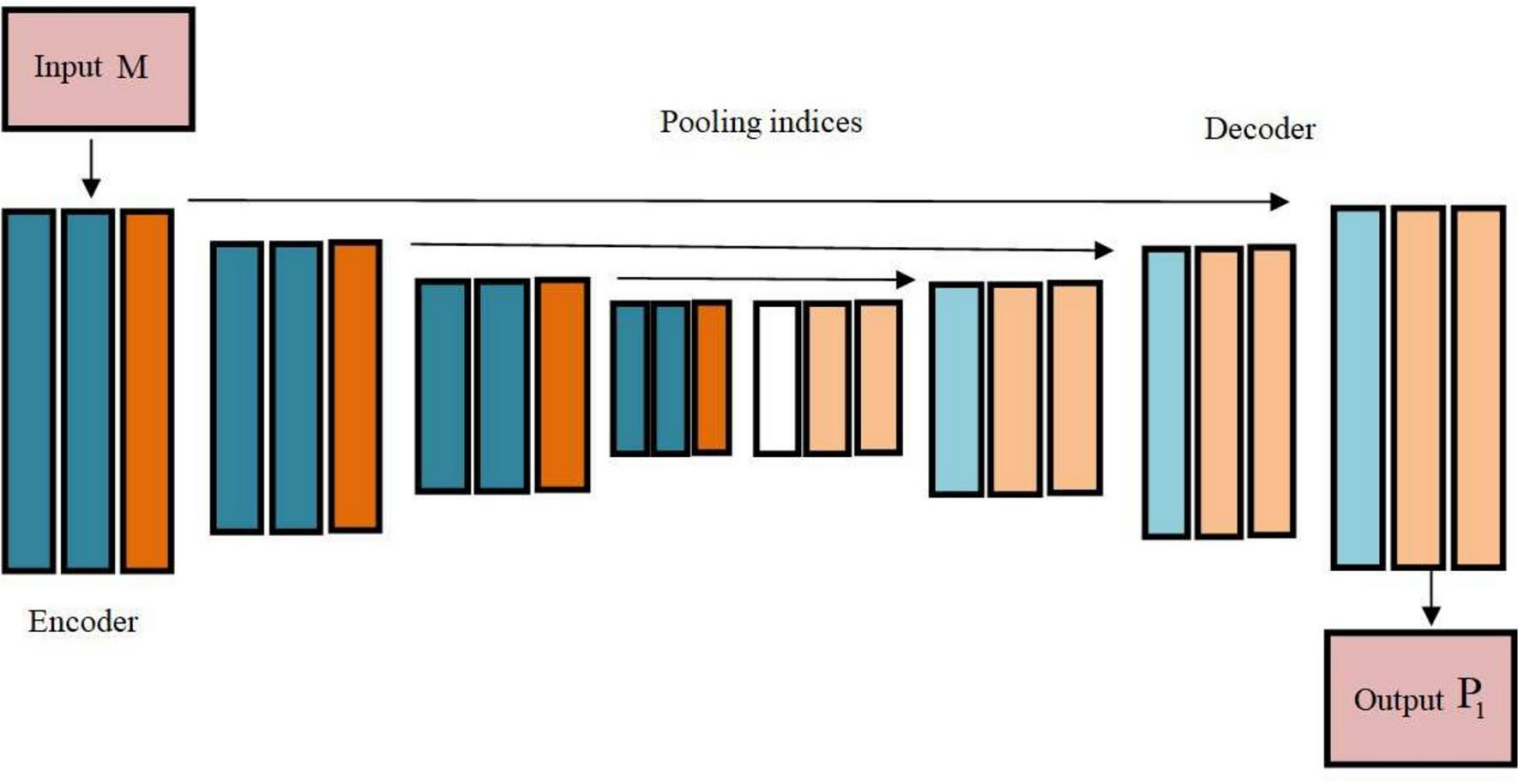
Architecture of SegNet.

SegNet^20^ refers to Deep Neural Network (DNN) which was initially introduced for segmenting the specific region from an image. Moreover, SegNet is regarded as DNN with 3 decoder and encoder depths, and its encoder layers are the same as convolutional (Conv) layers of VGG16 network. All the encoders^21^ in the encoder network perform Conv with a filter bank to form feature maps, and then batch normalization is carried out. Additionally, after the execution of element-wise Rectified Linear Unit (ReLU), max-pooling is performed, and the result is finally sub-sampled. To attain translation invariance in some spatial shifts of the input image, max-pooling is employed. In feature map, subsampling generates a large input image framework for all pixels. However, loss in feature map spatial resolutions exists, when subsampling and various max-pooling layers has the ability to produce translation invariances for robust classification. A decoder utilizes pooling indices from the corresponding encoder’s max-pooling for creating a segmentation mask. Moreover, to lessen complexity, a fully connected is eliminated, which reduces the quantity of encoder sector parameters. By employing learned max-pooling indices from equivalent encoder feature maps, appropriate decoder up-samples an input feature map. Feature maps are then convolved by employing a trainable decoder filter bank for creating feature maps. Following this, every specific feature map is fed to the process of batch normalization. Furthermore, a trainable softmax classifier is employed by passing a higher dimensional feature description from the previous decoder’s output to classify all pixels separately. Further, class weights are considered by median frequency with class imbalance, and then applied to pixel layer categorization to form a weighted cross-entropy loss function, and it is modeled as,

2 $$\begin{aligned} R=-\frac{1}{V} \sum _{r=1}^V \sum _{n=1}^x \xi _k-\zeta _r^n \log \left( \eta _r^n\right) \end{aligned}$$

where, total quantity of classes is symbolized as *x*, weight of class *c* is specified as $\xi _c$ , overall number of instances is signified as *V*, $\eta _r^n$ and $\varsigma _r^n$ indicates prediction and label in class *n*, and outcome attained from SegNet is implied as $P_1$. Architecture of SegNet is shown in Fig. 2.

#### Architecture of deep joint segmentation

DeepJoint segmentation^22^ is employed to find the best segment in terms of region similarity with distance adjusted between segmentation and deep points. Three stages, like region fusion, joining and generation of segmentation points are included in Deepjoint segmentation. An input image is split into grids, and in joining phase, pixels are combined in terms of mean and threshold. Next, region fusion is executed based on similarity among regions, and two constraints are related to recognize the mean, based on which the pixels in grids with less mean value are combined for identifying mapped points. The segmented points are determined by evaluating the best segments, and the evaluation is done by measuring a distance among deep points and segmentation. The process of DeepJoint segmentation is given below.

1. Grid formation: The input image is separated into various grids, and this grids from an image *M* is signified as,
  3 $$\begin{aligned} L=\{ L_1, L_2,..., L_b,...,L_i \} \end{aligned}$$
  where, overall quantity of grid is specified as *i*, and $L_b$ designates the $b^{th}$ grid.
2. Joining stage: Pixels in intra-grid points are combined by taking into account the mean and threshold. In grids, an average value of pixels is considered when assessing a mean value, and after the threshold value is set, mean value is developed. Moreover, threshold value is set to 1, and below expression is obtained to find the average value.
  4 $$\begin{aligned} L_b = \frac{\sum _{\lambda =1}^S \tau _\lambda }{S} \end{aligned}$$
  where, total amount of pixels in grid $L_b$ is indicated as *S*, pixel values connected to $L_b$ is specified as $\tau _\lambda$, and the equation for combining the pixels is written as,
  5 $$\begin{aligned} L_b = \frac{\sum _{\lambda =1}^S \tau _\lambda }{S} \pm \omega \end{aligned}$$
  Here, threshold is mentioned as $\omega$.
3. Region fusion stage: Region fusion process is utilized, which is formed by the assigned grids. Further, region similarity is recognized by employing region fusion, and it executed regarding similarity of pixel intensities considering two rules given below.
  - The value of mean mentioned by $I_b$ should be less than 3.
  - Single grid point is chosen for each grid.
  To determine mapped points, a region similarity is formed based on the above two rules. Here, region of similarity is formulated by,
  6 $$\begin{aligned} I_b = \frac{\sum _{\varpi =1}^\sigma \alpha _\varpi ^j}{P} \end{aligned}$$
  where, over-all amount of joined pixels is represented as *P*, joined pixels in joining stage is exemplified as $\alpha _\varpi ^j$, $\sigma$ implies pixel size, and merged grids known as mapped points are depicted as,
  7 $$\begin{aligned} K=\{ K_1, L_2,..., K_u,...,k_e \} \end{aligned}$$
  Here, $K_u$ exemplifies $u^{th}$ mapped point, and entire quantity of mapped points is implied as *e* ,
4. Deep point identification: These points are identified by combining missed pixels with mapped points. Missed pixels are those that remain in the image after joining stage, and pixels that do not appear in threshold boundary is denoted as and it is designated as,
  8 $$\begin{aligned} (\varepsilon )=\left\{ \Gamma _y\right\} ; 1<y \le \delta \end{aligned}$$
  where, entire count missed pixels is mentioned as $\delta$, and $\Gamma _y$ characterizes pixels missed at joining stage. Later, the mapped and missed pixels are utilized for estimating deep points, and it is represented as,
  9 $$\begin{aligned} \delta _{points} = \varepsilon + K_u \end{aligned}$$
5. Finding of optimum segments: An iterative approach is employed for identifying optimal segments from deep points. First, segmentation points represented by *F* are chosen randomly. Further, less distance is selected as new segmented point, and distance among segmented points is adjusted. Here, the lowest distance is signified as,
  10 $$\begin{aligned} \Upsilon ^{dis} = \sqrt{\sum _{u=1}^g (F_u - \delta _{points_u})^2} \end{aligned}$$
  where, amount of deep points is symbolized as *m*, and the outcome attained in DeepJoint segmentation is characterized as $P_2$.

#### Fusion based upon RV coefficient

The outcomes produced by DeepJoint segmentation $P_2$ and SegNet $P_1$ are combined by employing RV coefficient, and it is articulated as,

11 $$\begin{aligned} {E}=\left\{ \begin{array}{lr} {P}_1; & \text{ if } R V\left( {P}_1, {P}_2\right) =1 \\ \text{ majority } \text{ voting } \text{ based } \text{ salection; } & \text{ if } R V\left( {P}_1, {P}_2\right) \ne 1 \end{array}\right. \end{aligned}$$

RV coefficient^23^ measures the relation between two sets of variables, which is based on the principle of two sets of variables with perfect correlation. The expression of RV coefficient is written as,

12 $$\begin{aligned} R V(Z, U)=\frac{\left\langle {N}_{{Z}}, {N}_U\right\rangle }{\left\| {N}_{{Z}}\right\| \left\| {N}_V\right\| } \end{aligned}$$

where, variables are indicated as *Z* and *U*, inner products matrix is implied as $N_Z$ and $N_U$. Moreover, if $P_1$ and $P_2$ are not equal to 1, the nearest pixels in segmented outcome is considered otherwise majority of pixel count outcomes is selected in terms of voting, and final segmented output is exemplified as *E*.

### Image augmentation

Image augmentation is an effective model to reduce the error during the training process. This process is employed for enhancing the diversity of the given image, which is performed considering the outcome from segmented image *E*.

#### Geometric transformation

Geometric transformation^33^ is exploited as the mostly commonly used basic augmentation approach. The transformation parameters are randomly sampled or predefined. A similar transformation is applied to contours if the image includes one or more contours related to it.

1. **Shift scale:** In order to assist image translations, the shift scale translates all the pixel images in every direction. During training, shift scaling probabilities ranges from 0.7 to 0.9, and the shift scaled image is denoted as $J_1$.
2. **Rotate:** Rotating an image is the most frequently employed augmentation method. During this process, the information present in an image does not change even when it is rotated, and the rotated image is exemplified as $J_2$.
3. **Center crop flip:** The center cropping and flipping process is utilized to crop an image and flip it any directions, like right, down, left, or up. Here, center crop flipped image is designated as $J_3$.

#### Colorspace transformation

Photo-metric transformations is regarded as color space transformations^34^. Here, 3 stacked image matrix is introduced, and this matrix signifies pixel value for all red, green, and blue color values. Moreover, color distributions are adjusted for overcoming the lighting problems.

1. **Random brightness:** To enhance the quality of images, random brightness is employed, which is also utilized to create images with many brightness levels. Here, the random brightened image is denoted as $J_4$.
2. **Hue saturation:** It is employed for examining an alternative color for objects and circumstances in an image by arbitrarily varying the color channel, and hue saturated image is exemplified as $J_5$.

#### Mixup

The process of mixing the images is also considered as sample pairing, which is performed by randomly picking the second image, and it is overlaid over an original image for producing a new image. The new image after mixing is employed to train the classification process. Training samples are transformed into new samples, which is changes using two arbitrarily selected images from training data. This process is improved by creating a highly generalized version of mixing images. Moreover, in order to integrate the images, random cropping and patching are utilized, and the mixed images are designated as $J_6$.

#### Cutmix

The two images are combined together using CutMix and it produces new images with small quantity of pixels from an original image. This CutMix approach randomly selects the rectangular portions of one image, and extracts and pastes the image to another image. This method is done in terms of mask-enabled mixing, where the binary masks of similar sizes to the images are utilized to combine two of the images. Here, cutmix image is mentioned as $J_7$.

The resultant outcome produced from image augmentation is articulated as,

13 $$\begin{aligned} J \in \{ J_1, J_2,..., J_7 \} \end{aligned}$$

### Feature extraction

The dimensions in an image are reduced by employing feature extraction process, and it is utilized to detect typical features from an image. Here, augmented image *J* is fed as input for extracting redundant features. Moreover, features namely statistical, textural, LNDP, and DWT based LDP with entropy are mined.

#### Texture features

Various textural features, including correlation, contrast, homogeneity, and energy are employed for detecting skin cancer from the augmented image.

1. **Contrast:** Local differences in gray level co-occurrences matrix are adjusted by contrast^24^, and it is used to return the contrast intensity measure between every pixel and its nearest pixel from an image, and it is depicted as,
  14 $$\begin{aligned} \zeta _1 = \sum _{p,t} |p-t|^2 \gamma (p,t) \end{aligned}$$
  where, value of pixel at a specific location is denoted as $\gamma (p,t)$, $\zeta _1$ specifies contrast, and pixel coordinates are signified as *p* and *t*.
2. **Correlation:** The joint probability incidence of certain pixel pairings is regulated by using correlation^24^. It produces a pixel’s measure, which is connected to its nearest pixel over a whole image, and it varies from 1 to -1, and it is represented as,
  15 $$\begin{aligned} \zeta _2 = \sum _{p,t} \frac{(p-lp)(t-lt)\gamma (p,t)}{t_p t_t} \end{aligned}$$
  Here, $\zeta _2$ denotes correlation, *lp* and *lt* indicates mean, $t_p$ and $t_t$ specifies variance.
3. **Energy:** The sum of squared elements is provided by energy, and it is also known as uniformity. The value of energy varies from 0 to 1, but for a constant image, the value of energy is 1. The equation of energy^24^ is modeled as
  16 $$\begin{aligned} \zeta _3 = \sum _{p,t} \gamma (p,t)^2 \end{aligned}$$
  Here, $\zeta _3$ represents energy.
4. **Homogeneity:** It is used for measuring the value which is nearer to elements distribution, and values range from 0 to 1. Moreover, the homogeneity^24^ value is regarded as 1, and it is signified as,
  17 $$\begin{aligned} \zeta _4 = \sum _{p,t} \frac{\gamma (p,t)}{1+|p-t|} \end{aligned}$$
  where, $\zeta _4$ epitomizes homogeneity.

#### Statistical features

The statistical features, namely skewness, variance, and mean are utilized to identify skin cancer, and these features are explained below.

1. **Mean:** It^24^ represents the distribution of data concentration in the image, and the expression of mean is designated as,
  18 $$\begin{aligned} \zeta _5 = \frac{1}{m \times v} \sum _{p=0}^m \sum _{t=0}^v \gamma (p,t) \end{aligned}$$
  Here, size of grey level image is designated as $m \times v$ , and $\zeta _5$ represents mean.
2. **Variance:** The distribution of gray level in an image from mean gray level is termed as variance^24^. Here, variance is signified as,
  19 $$\begin{aligned} \zeta _6 = \frac{1}{m \times v} \sum _{p=0}^m \sum _{t=0}^v (\gamma (p,t) - \zeta _5)^2 \end{aligned}$$
  where, $\zeta _6$ indicates variance.
3. **Skewness:** It is designated as a process of measuring the asymmetry of distribution^24^, and it is expressed as,
  20 $$\begin{aligned} \zeta _7= & \frac{\sum _{y^\prime }^{e^\prime } (h_{e^\prime } - \overline{\zeta _5})}{(e^\prime - 1) \times N} \end{aligned}$$
  21 $$\begin{aligned} N= & \sqrt{\frac{1}{m \times v} \sum _{p=0}^m \sum _{t=0}^v (\gamma (p,t) - \zeta _5)^2} \end{aligned}$$
  where, $e^\prime$ indicates total images, $h_{e^\prime }$ signifies randomly chosen image, and $\zeta _7$ shows skewness.
  The combination all the features of statistical and textural features are designated as,
  22 $$\begin{aligned} Y= \{ \zeta _1, \zeta _2,..., \zeta _7 \} \end{aligned}$$

#### LNDP

LNDP^25^ is one of the latest feature extraction methods, which produces binary patterns to signify individual pixels in an image and mines local features in terms of nearest pixel differences. For the nearest pixels $W_o (o=1,2,....,8)$ of center pixel $W_r$ , LNDP is represented as,

23 $$\begin{aligned} d_1^o= & W_8 - W_\varepsilon , d_2^o = W_{o+1} - W_o, for o=1 \end{aligned}$$

24 $$\begin{aligned} d_1^o= & W_{8-1} - W_o, d_2^o = W_{o+1} - W_o, \forall o=2,3,..,7 \end{aligned}$$

25 $$\begin{aligned} d_1^o= & W_{8-1} - W_o, d_2^o = W_1 - W_o, for o=8 \end{aligned}$$

The change in specific neighboring pixels with two alternative nearest pixels is obtained in $d_1^o$ and $d_2^o$, and due to these differences, a binary number $A_3$ is assigned to each nearest pixel as follows.

26 $$\begin{aligned} A_3\left( d_1^o, d_2^o\right) =\left\{ \begin{array}{lllll} 1, & \text{ if } & d_1^o \ge 0 & \& & d_2^o \ge 0 \\ 1, & \text{ if } & d_1^o<0 & \& & d_2^o<0 \\ 0, & \text{ if } & d_1^o \ge 0 & \& & d_2^o<0 \\ 0, & \text{ if } & d_1^o<0 & \& & d_2^o \ge 0 \end{array}\right. \end{aligned}$$

Furthermore, LNDP is obtained by employing the above-mentioned binary values, and it is designated as,

27 $$\begin{aligned} \zeta _8 = \sum _{o=1}^8 2^{o-1} \times A_3 (d_1^o, d_2^o) \end{aligned}$$

Here, LNDP is mentioned as $\zeta _8$.

#### DWT based LDP with entropy

The augmented image *J* is passed to DWT, which is separated into four bands, like High–High (HH), Low–Low (LL), High–Low (HL), and Low–High (LH). Here, HH band is eliminated due to more noise. Following this, LL, LH and HL are given to LDP, which is then concatenated, and histogram is employed. Further, the entropy is measured to generate the DWT-based LDP with entropy feature.

- **DWT:** DWT^27^ is employed to mine the features from augmented images. It is utilized for mining the wavelet coefficients, and controls the frequency of images, which are vital for classification. Here, DWT is expressed as,
  28 $$\begin{aligned} \textrm{Z}\left( f^{\prime }\right) =\left\{ \begin{array}{l} t_{\mu , v^\prime }^{\prime }=\sum c^{\prime }\left( f^{\prime }\right) q^{\prime }*\mu \left( f^{\prime }-2 \mu v^\prime \right) \\ t_{\mu , v^\prime }^{\prime }=\sum c^{\prime }\left( f^{\prime }\right) j^{\prime }*\mu f^{\prime }i-2 \mu v^\prime ) \end{array}\right. \end{aligned}$$
  where, $v^\prime$ and $\mu$ implies translation factors and wavelet scale, $t^\prime _{\mu ,v^\prime }$ denotes element feature in $c^\prime (f^\prime )$ chosen to wavelet function, $q^\prime (f^\prime )$ and $j^\prime (f^\prime )$ exemplifies high-pass and low-pass filter coefficients.
- **LDP:** LDP^28^ is employed to measure the response of local direction around every pixel and then binary code is formed. Moreover, LDP is transmuted to decimal value, further edge responsive values are calculated in all directions.
  29 $$\begin{aligned} T = \sum _{\varphi =1}^7 q (\vartheta _\varphi - \vartheta _{x^\prime }) 2^\varphi \end{aligned}$$
  Here, *T* designates LDP, $\vartheta _{x^\prime }$ denotes kirsch activation, output from DWT based LDP is designated as *K*.
- **Entropy:** Entropy^26^ is used to illustrate the randomness of textural images, and it is written as,
  30 $$\begin{aligned} I^\prime = - \sum _{s^\prime =0}^{i^\prime -1} \sum _{p^\prime =0}^{b^\prime -1} K(s^\prime , p^\prime ) log_2 K(s^\prime , p^\prime ) \end{aligned}$$
  where, $I^\prime$ exemplifies entropy, $i^\prime \times b^\prime$ represents size of DWT based LDP, and DWT based LDP with entropy is specified as $\zeta _9$.
  Furthermore, by joining all extracted features, the final feature $\zeta$ is designated as,
  31 $$\begin{aligned} \zeta \in \{Y, \zeta _8, \zeta _9 \} \end{aligned}$$

### Skin cancer detection

**Fig. 3:**
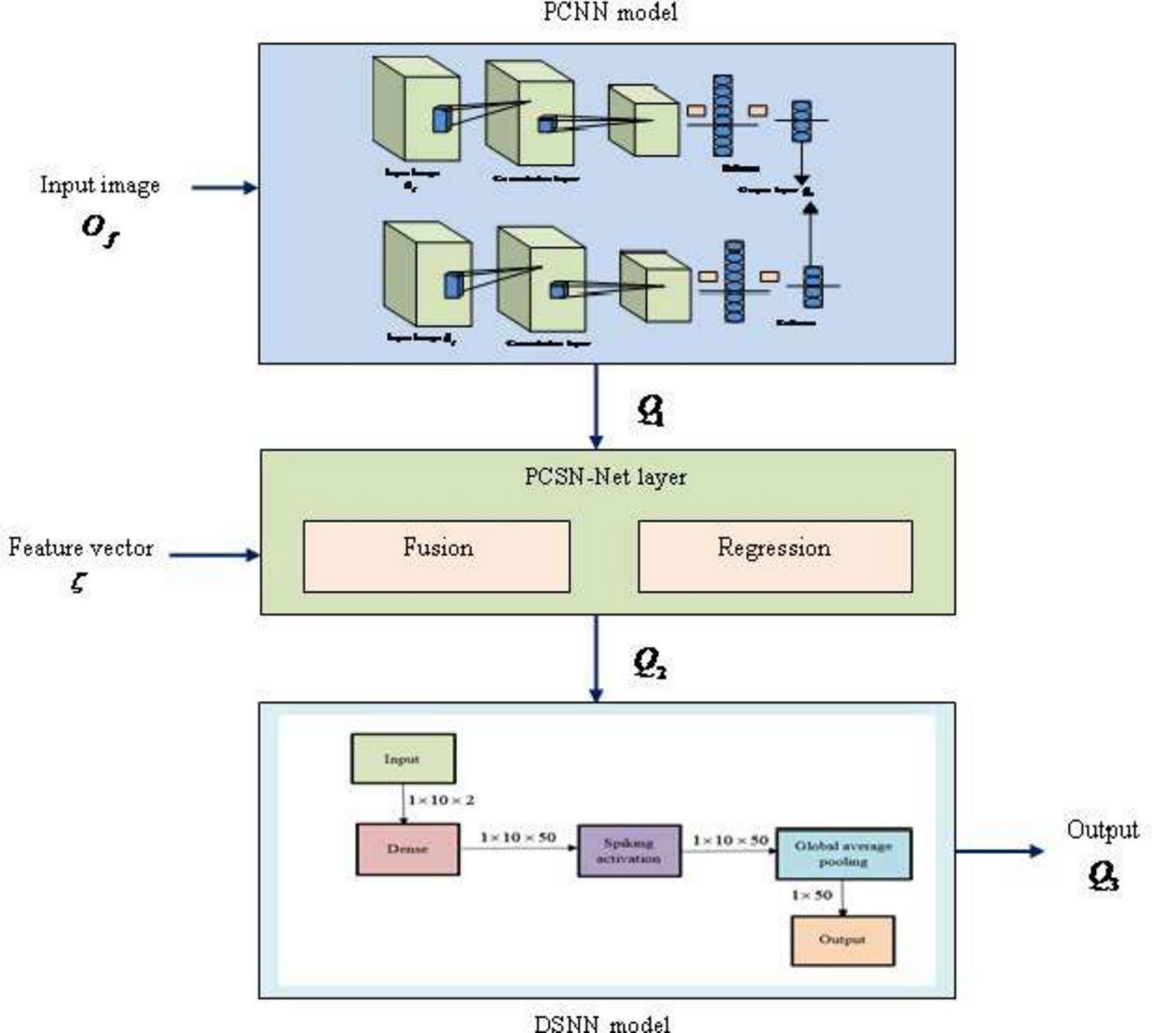
General structure of PCSN-Net for skin disease detection.

One of the deadliest kinds of cancer is skin cancer. This cancer is treatable when the disease is detected early as it is curable in its initial stage and if the disease is not detected it spreads gradually to other parts of body. Early detection of skin cancer is crucial due to its high death rate. Thus, a new technique, termed PCSN-Net is employed form detecting the skin cancer appropriately. Moreover, PCSN-Net is created by the integration of PCNN^29^ and DSNN^30,31^. The PCSN-Net comprises of three components, namely PCNN model, PCSN-Net layer, and DSNN model. Here, input image $O_f$ is taken as input to PCNN, and an output $Q_1$ is produced. Following this, $Q_1$ from PCNN and feature vector $\zeta$ is fed as input of PCSN-Net layer, where fusion is done based on regression modeling and thus $Q_2$ is attained. Furthermore, regression is executed concerning the relation between target and expected variables. The attained outcome $Q_2$ from PCSN-Net is fed as input to DSNN for attaining an output $Q_3$. General structure of PCSN-Net layer is shown in Fig. 3.

#### PCNN model

In PCNN^29^. the input image $O_f$ is taken as input, and PCNN is employed as the basis for parallelizing the CNN approaches using data parallelism. Stochastic Gradient Descent (SGD) is utilized for tuning CNN, and parallel tuning creates the similar results as sequential tuning. Moreover, the overlapping problems are efficiently decreased by PCNN approach. The two significant features of PCNN are (1) gradient parameters are pooled into large chunks and lessened toward each node by asynchronous communications; (2) gradient measurements are repeated in few fully-connected layers. A set of variables for representing data structures are given below. Here, quantity of output-input rows of neutron is specified as $1_{out} / 1_{in}$ , output and input feature maps are signified as $h_{out} / h_{in}$, entire row and column filters are indicated as $1_{fil} / \Im _{fil}$, quantity of output-input columns are represented as $\Im _{out} / \Im _{in}$, quantity of neutron in bottom and current layers is implied as $K_s^\prime , K_w^\prime$, and quantity of images is characterized as *X*.

The Conv and fully-connected layers are the two kinds of computationally exhaustive layers in CNN. The computation pattern is altered from Conv layer to matrix multiplication and thus *image*2*Col* in Conv layer employed for reallocating the input data. Moreover, matrix multiplication is referred as computation pattern in fully connected layers, and hence entire computational workload is considered as a group of matrix multiplication and data transformation.

Here, the data transformation functions, like *Col*2*image* and *image*2*Col* are employed in open-source structures. A filter size of input image is transferred by *image*2*col* into columns, and then they are merged to make a two-dimensional matrix. Moreover, the column is relocated to the blocks of original data layout by *col*2*image* function. These models are precisely considered to alter massive quantity of images in input activation into single large matrix.

**Fig. 4:**
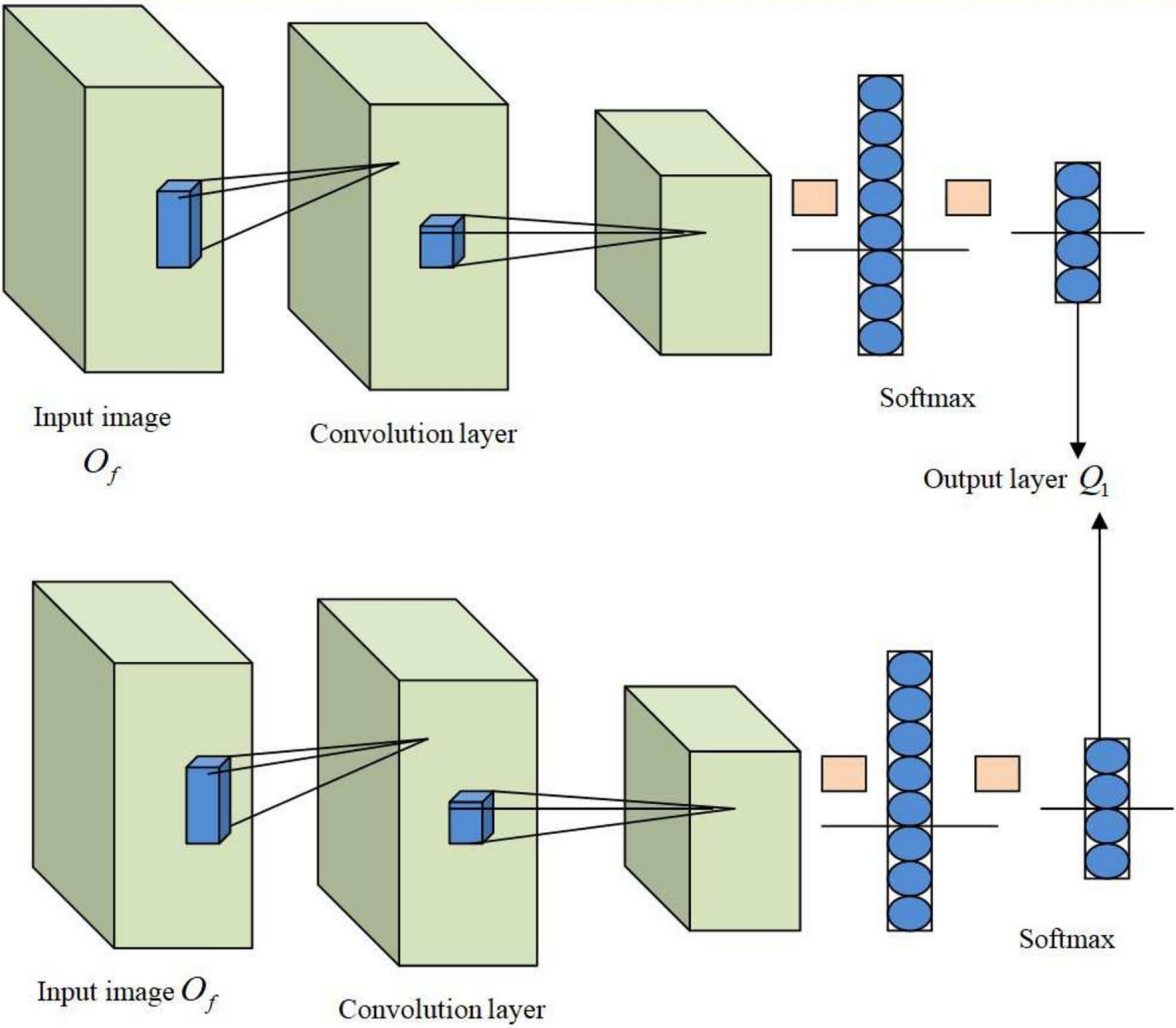
Architecture of PCNN.

In feed-forward, an input saved in major order in a row is considered as an input for Conv layer. Here, all mini batch of *x* image is created into dimension matrix $X \times h_{in} 1_{in} \Im _{in}$, and it is transmuted by *image*2*col* into $h_{in} 1_{fil} \Im _{fil} \times X 1_{out} \Im _{out}$ matrix. After this, matrix is multiplied by weight matrix $h_{out} \times h_{in} 1_{fil} 1_{out}$ and added to bias vector $h_{out}$, and outcome of activation matrix $h_{out} \times h_{out} 1_{out} 1_{out}$ is represented as,

32 $$\begin{aligned} C^{\prime v-1}= & image2col(\beta ^{v-1}) \end{aligned}$$

33 $$\begin{aligned} Q_1= & \wp (M^{\prime \prime n^\prime } O_f + \psi ^v \end{aligned}$$

here, activation matrix layer *v* is indicated as $\beta ^v$, activation function is mentioned as $\wp$ , matrix created by *image*2*col* is implied as $C^\prime$, $\psi$ exemplifies bias. In Conv layer, input from before layer is dimension of activation matrix $h_{in} \times B 1_{in} \Im _{in}$, generation of *image*2*col* for both layouts is denoted as $B \times h_{in} 1_{in} \Im _{in}$ and $h_{in} \times B 1_{in} \Im _{in}$, and $Q_1$ signifies the output of PCNN layer. The Fig. 4 represents PCNN structure.

#### PCSN-Net layer

The output $Q_1$ from PCNN and feature vector is taken as input for PCSN-Net layer. Here, the fusion of the output from PCNN and mined features in terms of regression is performed by using Fractional Calculus (FC)^35^. Moreover, fusion is obtained by contemplating the outcome at different time instances. Furthermore, PCSN-Net produces an outcome $Q_2$, and it is modeled as follows,

At time *D*, output is accomplished by considering the statistical and textural features. Here, total of 7 statistical and textural features are utilized, thus size ranges from 1 to 7, and the output is expressed as,

34 $$\begin{aligned} D = \sum _{k=1}^7 Y_k * I_k \end{aligned}$$

At time $(D-1)$ , the outcome attained by considering LNDP feature is specified as $D_1$, and it is modeled as,

35 $$\begin{aligned} D_1 = \sum _{k=1}^{u^\prime } \sum _{h^\prime =1}^{d^\prime } \zeta _{8_k} * I_k \end{aligned}$$

here, dimension of LNDP is specified as $u^\prime$ and $d^\prime$, and $I_k$ characterizes weight. At time $(D-2)$, the outcome produced by considering DWT based LDP with entropy is designated as $D_2$, and it is depicted as,

36 $$\begin{aligned} D_2 = \sum _{k=1}^{u^\prime } \zeta _{9_k} * I_k \end{aligned}$$

In order to enhance the computation performance by lessening the information loss FC is employed. Moreover, the general expression of FC^35^ is represented as,

37 $$\begin{aligned} \textrm{D}(D+1)=\Psi \textrm{D}(D)+\frac{1}{2} \Psi \textrm{D}(D-1)+\frac{1}{6}(1-\Psi ) \textrm{D}(D-2)+\frac{1}{24}(1-\Psi )(2-\Psi ) \textrm{D}(D-3) \end{aligned}$$

By applying Eqs. (34–36) in Eq. (37), we get

38 $$\begin{aligned} Q_2=\Psi * \sum _{k=1}^7 Y_k * I_k+\frac{1}{2} \Psi * \sum _{k=1}^{u^{\prime }} \sum _{h^\prime =1}^{d^\prime } \zeta _{8_k}+I_k+\frac{1}{6}(1-\Psi ) * \sum _{k=1}^{u^{\prime }} \zeta _{9_k}*I_k+\frac{1}{24}(1-\Psi )(2-\Psi ) Q_1 \end{aligned}$$

By applying $Q_1$ from Eq. (33), we get

39 $$\begin{aligned} Q_2=\Psi * \sum _{k=1}^7 Y_k * I_k+\frac{1}{2} \Psi * \sum _{k=1}^{u^{\prime }} \sum _{h^\prime =1}^{d^\prime } \zeta _{8_k}+I_k+\frac{1}{6}(1-\Psi ) * \sum _{k=1}^{u^{\prime }} \zeta _{9_k}*I_k+\frac{1}{24}(1-\Psi )(2-\Psi ) * \wp (M^{\prime \prime n^\prime } O_f + \psi ^v) \end{aligned}$$

here, $\Psi$ postulates constant, statistical and textural features are epitomized as $Y_k$, LNDP feature is denoted as $\zeta _8$, DWT based LDP with entropy is implied as $\zeta _9$, $Q_2$ designates the output from PCNN, and $I_k$ epitomizes weight.

#### DSNN model

DSNN is Artificial Neural network which closely imitates neural networks, here deep states entire network and spiking states activation of single neuron. This DSNN is employed as it is computationally efficient and highly robust. The input layer neurons in this network produces signed spikes or spikes with equivalent positive or negative value, by utilizing a method termed as Spiking Vector Quantization^30,31^. A real vector *z* is applied which represents the input to a collection of neurons with time-steps *G*, and generates a sequence of $P^\prime$ signed-spikes, such as $<(\left( g_v^{\prime } l_v^{\prime }\right) : g^{\prime } \in [1 \operatorname {len}(z)], l^{\prime } \in \{ \pm 1\}, v \in [1..P^{\prime } ]>$. Here, index of neuron from $v^{th}$ spike fires is mentioned as $g_v^\prime$, entire sum of spikes produced is indicated as $P^\prime$ , and $l_v^\prime \in {\pm 1}$ implies the sign of $v^{th}$ spike.

Moreover, at time step *G*, input spikes to neuron $a^\prime$ at layer *E* are combined by the below equation,

40 $$\begin{aligned} w_{a^\prime }^E (G) = \sigma ^\prime \sum _{n^\prime } U_{a^\prime n^\prime }^{\prime E-1}. Q_2 \end{aligned}$$

here, $Q_2$ denotes output from PCSN-Net layer, neuron firing threshold is exemplified as $\sigma ^\prime$ , synaptic weight which joins afferent neuron $n^\prime$ from $E-1$ layer is signified as $U_{d n^\prime }^ {\prime E-1}$. Further, input current $w_{a^\prime }^E (G)$ is integrated by neuron $a^\prime$ into membrane potential $L_{a^\prime }^{\prime E} (G)$, and $L_{a^\prime }^{\prime E} (G)$ is initialized with learnable parameter $O_{a^\prime }^\prime$, and spike output is created when $L_{a^\prime }^{\prime E} (G)$ crosses the firing threshold $\sigma ^\prime$, which is depicted as,

41 $$\begin{aligned} & L_{a^\prime }^{\prime E} (G) = L_{a^\prime }^{\prime E} (G-1) + w_{a^\prime }^E (G) - \sigma ^\prime . \varphi _{a^\prime }^E (G-1) \end{aligned}$$

42 $$\begin{aligned} & L_{a^\prime }^{\prime E} (0) = O_{a^\prime }^\prime \end{aligned}$$

43 $$\begin{aligned} & \varphi _{a^\prime }^{E}(G)=\Theta \left( L_{a^\prime }^{\prime \textrm{E}}(G)-\sigma ^{\prime }\right) \text{ with } \Theta (x)=\left\{ \begin{array}{l} 1, \text{ if } x \ge 0 \\ 0, \text{ otherwise } \end{array}\right. \end{aligned}$$

The overall quantity of spikes created by neuron $n^\prime$ at input layer is identified by summing every incoming spikes at time *G*. Moreover, raw intensity values sums from a Poisson generator is obtained as input.

44 $$\begin{aligned} w_{a^\prime }^E = \sigma ^\prime \sum _{n^\prime } U_{a^\prime n^\prime }^{\prime E-1}. Q_2 + O_{a^\prime }^\prime \end{aligned}$$

**Fig. 5:**
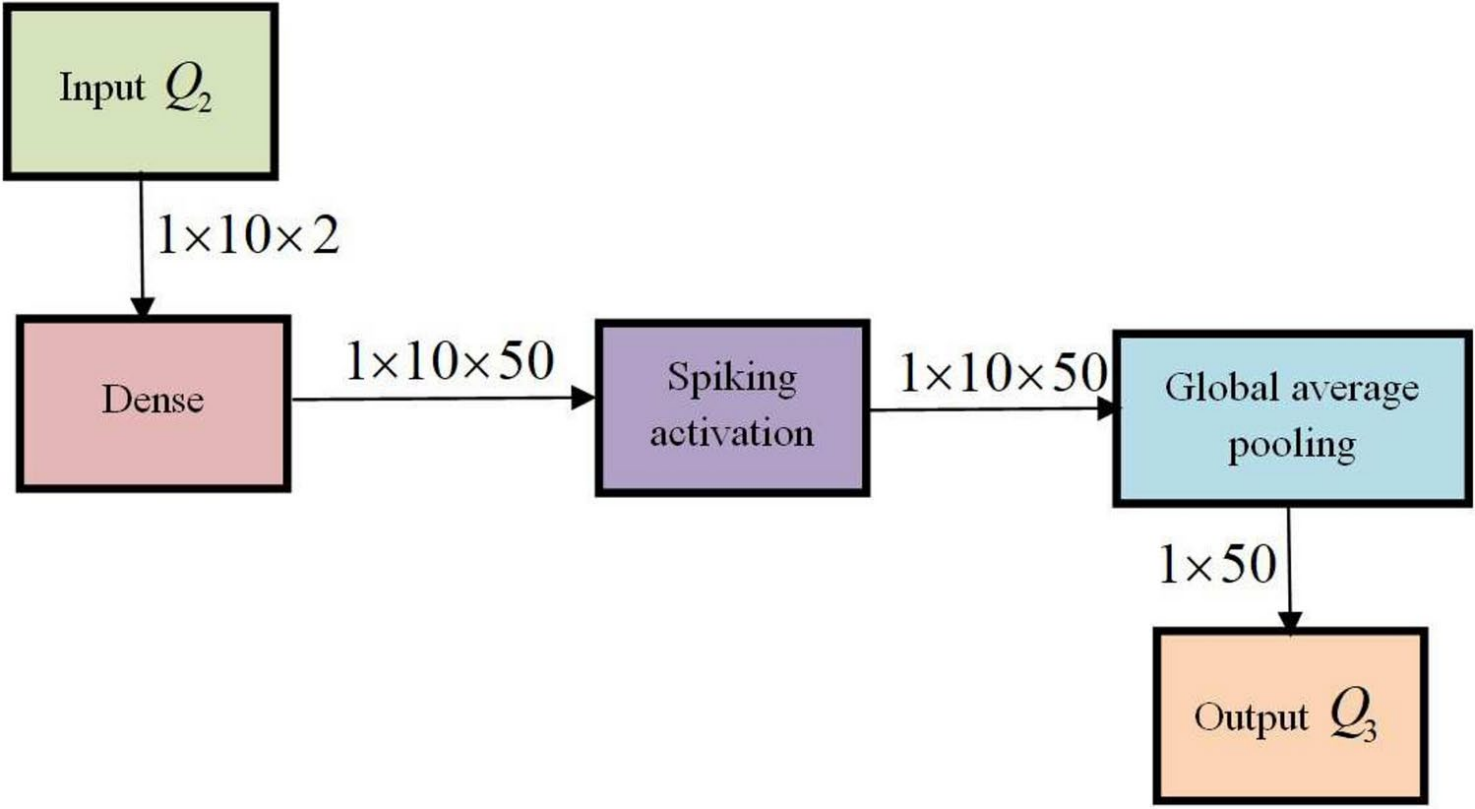
Architecture of DSNN.

By substituting Eq. (40) in Eq. (41), we get,

45 $$\begin{aligned} Q_3= & L_{a^\prime }^{\prime E} (G-1) + \sigma ^{\prime } \sum _{n^\prime } U_{a^\prime n^\prime }^{\prime E-1}. Q_2 - \sigma ^\prime . \varphi _{a^\prime }^E (G-1) \end{aligned}$$

46 $$\begin{aligned} Q_3= & L_{a^\prime }^{\prime E}(G-1)+\sigma ^{\prime } \sum _{n^\prime } U_{a^\prime n^\prime }^{\prime E-1}. (\Psi * \sum _{k=1}^7 Y_k * I_k+ \frac{1}{2} \Psi * \sum _{k=1}^{u^{\prime }} \sum _{h^\prime =1}^{d^\prime } \zeta _{8_k}+I_k+ \frac{1}{6}(1-\Psi ) * \sum _{k=1}^{u^{\prime }} \zeta _{9_k}*I_k \nonumber \\ & + \frac{1}{24}(1-\Psi )(2-\Psi ) * \wp (M^{\prime \prime n^\prime } O_f + \psi ^v)) - \sigma ^\prime . \varphi _{a^\prime }^E (G-1) \end{aligned}$$

here, $Q_3$ specifies the outcome of DSNN, and Fig. 5 illustrates the architecture of DSNN.

## Results and discussion

The PCSN-Net approach novelty comes from its unusual design, that combines a Parallel Convolutional Neural Network (PCNN) and a Deep Spiking Neural Network (DSNN). This combination addresses numerous major issues in skin cancer detection. One major problem in present techniques is attaining high precision in discriminating between malignant and benign lesions but preventing overfitting because of insufficient information. The proposed framework addresses this issue using modern methods for data enhancement such as geometric transformations, Mixup, and CutMix, that enhance information diversity while reducing overfitting. Another problem is obtaining delicate textural and statistical aspects of skin lesions, which are frequently ignored by typical CNNs. PCSN-Net’s feature extraction stage includes Discrete Wavelet Transform (DWT)-based Local Direction Patterns (LDP) and Local Normal Derivative Patterns (LNDP), which enable extensive study of skin lesion structures. This strategy improves the model’s sensitiveness and specificity, enabling it to identify tiny differences that are important for discriminating between lesion forms. Furthermore, the use of a Deep Spiking Neural Network (DSNN) meets the demand for energy efficiency, which is a significant improvement over traditional deep learning models. The DSNN’s sparse, event-driven calculations make it ideal for real-time applications, which are critical in medical applications with restricted computing resources. By combining accuracy and computing economy, PCSN-Net provides a novel strategy for early skin cancer detection that is both dependable and feasible, overcoming fundamental shortcomings in previous systems. The experimental outcomes obtained by PCSN-Net used for skin cancer detection identifies the superiority of PCSN-Net by comparing its performance with traditional skin cancer detection approaches are listed below,

### Experimentation setup

The PCSN-Net model used for skin cancer detection is implemented using a Python tool by utilizing Skin Cancer: Malignant vs. Benign dataset^32^.

### Description of dataset

This dataset is available under open-source licensing for non-commercial use, which is accessible to researchers and developers for testing various skin cancer detection algorithms. The database comprises about 1800 skin images of $244 \times 244$ size taken from two types of moles. Skin Cancer: Malignant vs. Benign dataset^9^, which was utilized to develop the PCSN-Net model, is publicly accessible on Kaggle. It contains numerous labeled dermoscopic photos of skin lesions classified as malignant (cancerous) or benign (non-cancerous). This dataset is frequently employed in medical research to build and test skin cancer detection methods. The dataset includes high-resolution photos that enable for extensive examination of textural, color, and structural patterns, all of which are critical in identifying malignant from non-cancerous tumors. Its balanced classes and range of lesion types make it ideal for training deep learning models such as PCSN-Net, since it provides real-world variability that increases model generalizability and robustness. Furthermore, the dataset includes information, such as lesion location and patient demographics, which can help in further analysis if required.

### Performance measures

In order to determine the performance of PCSN-Net, different performance metrics are utilized and are delineated below,

- **Accuracy:** Accuracy is utilized to identify the relationship between actual and expected output determined by PCSN-Net, which is expressed as.
  47 $$\begin{aligned} Accuracy = \frac{O_{TP} + O_{TN}}{O_{TP} + O_{TN} + O_{FP} + O_{FN}} \end{aligned}$$
  here, $O_{TN}$ and $O_{TP}$ resembles true negative and true positive, false negative and false positive is represented as $O_{FN}$ and $O_{FP}$.
- **Specificity:** The ratio of correctly detected negative labels by PCSN-Net from total negative labels taken as input is termed specificity. The specificity is expressed by,
  48 $$\begin{aligned} Specificity = \frac{O_{TN}}{O_{TN} + O_{FP}} \end{aligned}$$
- **Sensitivity:** The ratio of correctly detected positive labels by PCSN-Net from total positive labels taken as input is termed sensitivity, which is given as,
  49 $$\begin{aligned} Sensitivity = \frac{O_{TP}}{O_{TP} + O_{FN}} \end{aligned}$$
- **NPV:** The ratio of correctly detected negative labels from the total detected negative label by PCSN-Net is termed NPV, which is given by,
  50 $$\begin{aligned} NPV = \frac{O_{TN}}{O_{FN} + O_{TN}} \end{aligned}$$
- **PPV:** The ratio of accurately detected positive labels from the total detected positive label by PCSN-Net is PPV which is given by the expression.
  51 $$\begin{aligned} PPV = \frac{O_{TP}}{O_{TP} + O_{FP}} \end{aligned}$$
- **FPR:** FPR is the proportion of total negative samples that are incorrectly detected as positive samples by PCSN-Net. The FPR is formulated as,
  52 $$\begin{aligned} FPR = \frac{O_{FP}}{O_{FP} + O_{TN}} \end{aligned}$$

### Experimentation results

**Fig. 6:**
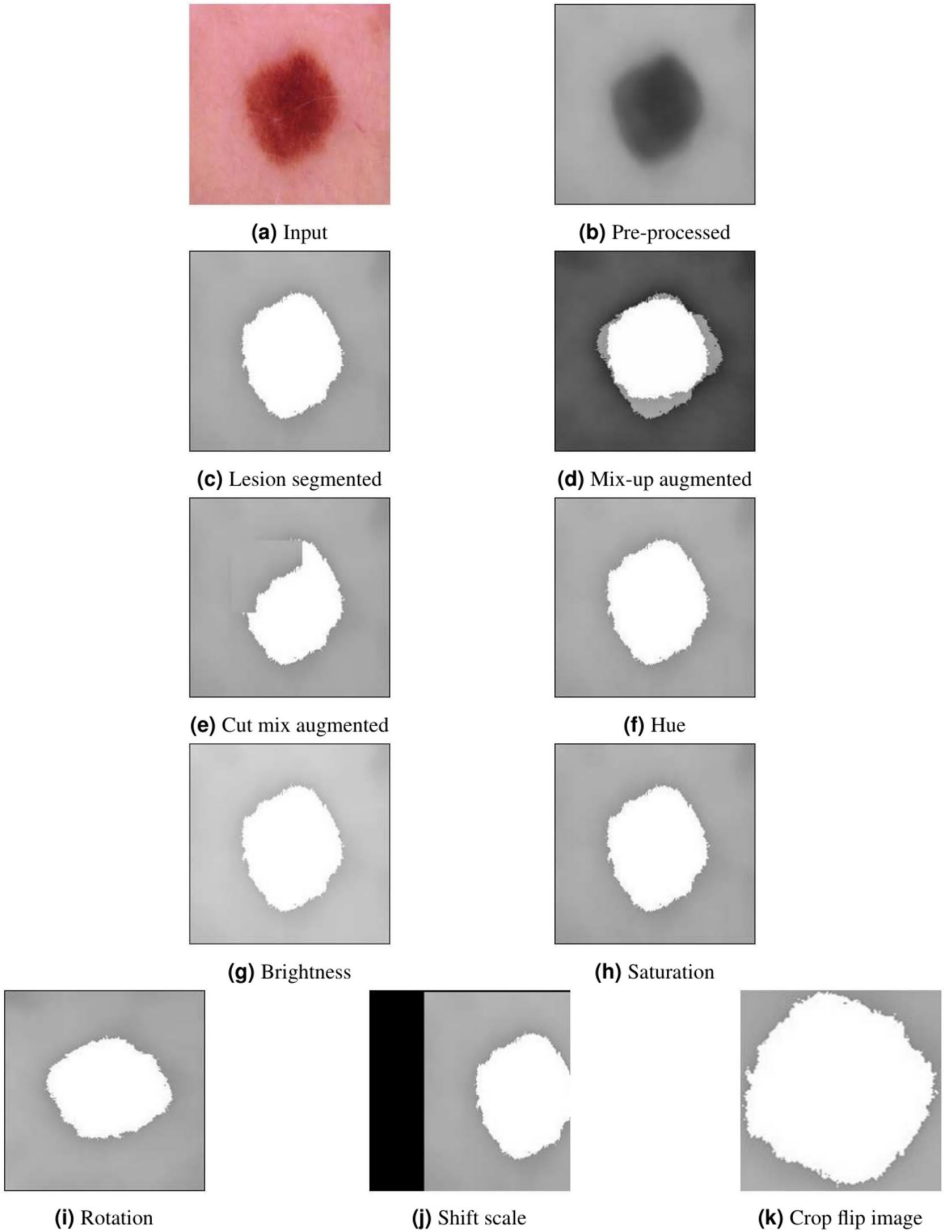
Image results of PCSN-Net.

The image outcomes obtained from PCSN-Net are displayed in Fig. 6, where the input and resultant filtered image are shown in Fig. 6a,b. The segmented skin cancer image is given in Fig. 6c, and the mix-up as well as cut mix image is elucidated in Fig. 6d,e. Moreover, the colorspace transformation image, like hue, brightness, and saturation skin lesion image is shown in Fig. 6f–h. Also, the resultant geometric transformation image, like rotation, crop flip, and shift scale image is depicted in Fig. 6i–k.

### Performance evaluation

**Fig. 7:**
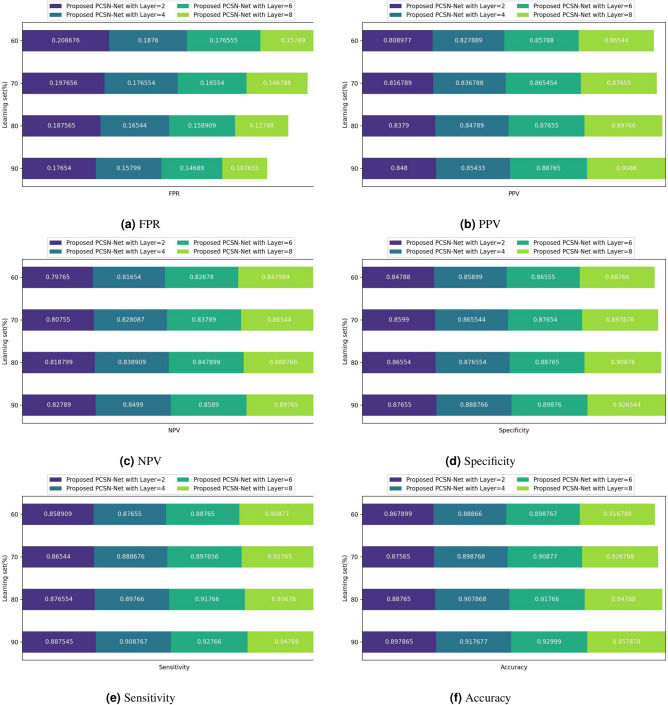
Performance validation of PCSN-Net employing.

The performance validation of PCSN-Net used for detecting skin cancer is illustrated in Fig. 7. The validation of PCSN-Net using FPR is given in Fig. 7a, where the PCSN-Net obtained FPR of 0.176, 0.157, 0.146, and 0.107 for the learning set of 90% and for 2,4,6, and 8 layers. Figure 7b shows the analysis of PCSN-Net used to identify skin cancer using PPV. Here, for learning set of 90%, the PCSN-Net obtained PPV of 0.848, 0.854, 0.887, and 0.908 for layers 2,4,6, and 8. The evaluation of PCSN-Net used to detect skin cancer from skin image based on NPV is displayed in Fig. 7c. Here, the NPV observed by PCSN-Net for 2,4,6, and 8 layers is 0.827, 0.849, 0.858, and 0.897, for learning set of 90%. Furthermore, Fig. 7d depicts the performance analysis of PCSN-Net by utilizing specificity. Here, PCSN-Net observed specificity of 0.876, 0.888, 0.898, and 0.926 for learning set of 90% and for 2,4,6, and 8 layers. Further, the validation of PCSN-Net used for detecting skin cancer through sensitivity is depicted in Fig. 7e. The PCSN-Net measured 0.887, 0.908, 0.927, and 0.947 of sensitivity for 90% learning set and for 2,4,6, and 8 layers. In Fig. 7f, the validation of performance of PCSN-Net using accuracy is validated, where the PCSN-Net obtained accuracy of 0.897, 0.917, 0.929, and 0.957 for 90% learning set and for 2,4,6, and 8 layers.

### Comparative techniques

The superiority of PCSN-Net used for the detection of skin cancer is determined by comparing with existing detection models, like Aquila Whale Optimization-SqueezeNet (AWO-SqueezeNet)^36^, deep learning^6^, Ensemble lightweight deep learning^9^, Optimized CNN^4^, transfer learning^3^, and DeepSegNet+Fuzzy cognitive map (FCM).

### Comparative analysis

**Fig. 8:**
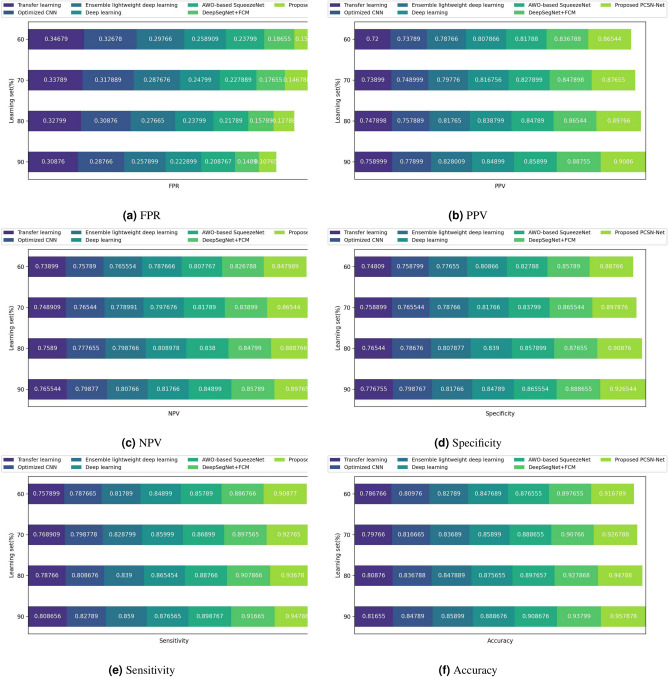
Validation of PCSN-Net employing.

Figure 8 delineates the analysis carried out to identify the performance of PCSN-Net for detecting skin cancer by varying learning set. Figure 8a shows the analysis of different approaches used for diagnosing skin cancer using FPR. The minimum FPR of 0.107 is observed for 90% learning set by PCSN-Net, where the other existing models recorded FPR of 0.208 by AWO-SqueezeNet, 0.222 by deep learning, 0.257 by Ensemble lightweight deep learning, 0.287 by Optimized CNN, 0.308 by transfer learning, and 0.148 by DeepSegNet+ FCM. The validation of different approaches used for detecting skin cancer utilizing PPV is demonstrated in Fig. 8b. Here, the PCSN-Net observed PPV of 0.858 and the PPV obtained by existing models, like AWO-SqueezeNet is 0.858, deep learning is 0.848, Ensemble lightweight deep learning is 0.828, Optimized CNN is 0.778, transfer learning is 0.758, and DeepSegNet+ FCM is 0.887 for learning set of 90%. The PCSN-Net obtained superior performance of 5.46% than existing AWO-SqueezeNet approach. The evaluation of various skin cancer detection models using NPV is depicted in Fig. 8c, where the NPV recorded by PCSN-Net designed in this research is 0.897 for 90% learning set. Similarly, the prevailing models used for detection, namely AWO-SqueezeNet, deep learning, Ensemble lightweight deep learning, Optimized CNN, transfer learning, and DeepSegNet+ FCM measured NPV of 0.848, 0.817, 0.807, 0.798, 0.765, and 0.857. The PCSN-Net attained high performance of 4.09% than the baseline DeepSegNet+FCM model. Figure 8d investigates the specificity recorded by the detection techniques used for identifying skin cancer. Here, the PCSN-Net attained maximum specificity of 0.926 than other baseline models Likewise, the existing models obtained specificity of 0.865 by AWO-SqueezeNet, 0.847 by deep learning, 0.817 by Ensemble lightweight deep learning, 0.798 by Optimized CNN, 0.776 by transfer learning, and 0.888 by DeepSegNet+ FCM for learning set of 90%. It is proven that the PCSN-Net obtained superior performance of 13.79% than Optimized CNN model. Furthermore, Fig. 8e shows the comparative assessment of various approaches used to detect skin cancer employing sensitivity. The sensitivity measured by PCSN-Net is 0.947 for 90% learning set and the prevailing approaches, like AWO-SqueezeNet, deep learning, Ensemble lightweight deep learning, Optimized CNN, transfer learning, and DeepSegNet+ FCM measured sensitivity of 0.898, 0.876, 0.859, 0.827, 0.808, and 0.916. Here, the superior performance of 5.18% is obtained by PCSN-Net than the prevailing AWO-SqueezeNet technique. In addition, Fig. 8f displays the validation of different approaches utilized to identify skin cancer from skin images using accuracy. The accuracy of 0.957 is observed by the designed PCSN-Net model, where the baseline models measured accuracy of 0.908, 0.888, 0.858, 0.847, 0.816, and 0.937 for learning set of 90% by AWO-SqueezeNet, deep learning, Ensemble lightweight deep learning, Optimized CNN, transfer learning, and DeepSegNet+ FCM. It is revealed that the PCSN-Net obtained maximum accuracy of 10.32% than the prevailing Ensemble lightweight deep learning approach r.

### Comparative discussion

The results recorded by PCSN-Net as well as the prevailing detection schemes are portrayed in Table 2. Here, the PCSN-Net obtained high performance with FPR of 0.107, PPV of 0.908, NPV of 0.897, sensitivity of 0.947, specificity of 0.926, as well as accuracy of 0.957 for 90% learning set. Similarly, the prevailing techniques, such as AWO-SqueezeNet, deep learning, Ensemble lightweight deep learning, Optimized CNN, transfer learning, and DeepSegNet+ FCM obtained FPR of 0.208, 0.222, 0.257, 0.287, 0.308, and 0.148, and PPV of 0.858, 0.848, 0.828, 0.778, 0.758, and 0.887. Moreover, the NPV obtained by existing models is 0.848 by AWO-SqueezeNet, 0.817 by deep learning, 0.807 by Ensemble lightweight deep learning, 0.798 by Optimized CNN, 0.765 by transfer learning, and 0.857 by DeepSegNet+ FCM. Also, the prevailing models obtained 0.865, 0.847, 0.817, 0.798, 0.776, and 0.888 of specificity for AWO-SqueezeNet, deep learning, Ensemble lightweight deep learning, Optimized CNN, transfer learning, and DeepSegNet+ FCM, where these techniques also recorded sensitivity of 0.898, 0.876, 0.859, 0.827, 0.808, and 0.916. The accuracy observed by AWO-SqueezeNet, deep learning, Ensemble lightweight deep learning, Optimized CNN, transfer learning, and DeepSegNet+ FCM is 0.908, 0.888, 0.858, 0.847, 0.816, and 0.937. The experimental results revealed that the PCSN-Net achieved high performance and is good enough in diagnosing skin cancer from skin images. The designed model accurately identifies skin cancer by performing extensive augmentation approaches and extracting discriminative features to obtain impressive results. Also, the PCSN-Net outperforms other existing techniques used for comparison by converging quickly and effectively handling overfitting problems.

**Table 2:** Comparative discussion.

Evaluation measure	DeepSegNet +FCM	AWO based SqueezeNet	Deep learning	Ensemble lightweight deep learning	Optimized CNN	Transfer learning	Proposed PCSN-Net
FPR	0.148	0.208	0.222	0.257	0.287	0.308	**0.107**
NPV	0.857	0.848	0.817	0.807	0.798	0.765	**0.897**
Accuracy	0.937	0.908	0.888	0.858	0.847	0.816	**0.957**
PPV	0.887	0.858	0.848	0.828	0.778	0.758	**0.908**
Specificity	0.888	0.865	0.847	0.817	0.798	0.776	**0.926**
Sensitivity	0.916	0.898	0.876	0.859	0.827	0.808	**0.947**

Significant values are in bold.

### Comparison of PCSN-Net with standard models

To validate the PCSN-Net model’s usefulness, compared with advanced techniques such as EfficientNet, DenseNet, ResNet with CBAM, Xception, Inception-ResNet-V2, Alex Net, VGG-16 and ResNet-50 demonstrate its benefits in skin cancer diagnosis. The PCSN-Net obtains 95.7% accuracy, outperforming EfficientNet and DenseNet, which have accuracy rates of roughly 93.5% and 94.0%, respectively. ResNet with CBAM and Inception-ResNet-V2, noted for their high accuracy because of attention mechanisms and hybrid designs, achieve precision of 94.5% and 95.0%, respectively, but fall somewhat short of PCSN-Net’s efficiency. In terms of sensitivity, PCSN-Net scores 94.7%, suggesting a strong capacity to correctly recognize malignant instances, which can be critical for medical use. This is slightly higher than the sensitivity rates of Xception and ResNet with CBAM, which are approximately 92.5% and 94.0%, correspondingly. PCSN-Net also has 92.6% specificity, indicating a good capacity to effectively classify benign instances, hence reducing false positives. While training times vary, with PCSN-Net taking approximately 2 hours on a single GPU, models such as DenseNet and Inception-ResNet-V2 typically take longer (2.5–3.5 hours) because to their deeper architectures and complicated layer connections. PCSN-Net also has efficient inference times (about 20 ms per image), making it appropriate for real-time diagnostic applications, unlike some other models, that might deliver a little slower inference due to additonal layers or attention mechanisms. Overall, PCSN-Net’s better combination of accuracy, sensitivity, specificity, and efficiency demonstrates legitimacy as an enhanced framework for skin cancer diagnosis, with higher accuracy and practical applicability. Table 3 shows the comparison of the proposed with other standard models.

**Table 3:** The comparison with the standard models.

Model	Accuracy	Sensitivity	Specificity	F1-score	Training time	Inference time	Advantages	Limitations
PCSN-Net (Proposed)	0.957	0.947	0.926	0.927	$\tilde{2}$ h (on single GPU)	$\tilde{2}0$ ms per image	High accuracy, energy-efficient	Computation-ally intensive
EfficientNet	0.935	0.92	0.91	0.915	$\tilde{1}.5$ h	$\tilde{1}5$ ms per image	Efficient, low computational cost	May require fine-tuning on medical images
DenseNet	0.94	0.93	0.915	0.922	$\tilde{2}.5$ h	$\tilde{3}0$ ms per image	Fast convergence, high accuracy	Increased memory requirement
ResNet + CBAM	0.945	0.94	0.92	0.93	$\tilde{3}$ h	$\tilde{2}5$ ms per image	Enhanced focus on relevant features	Higher computational cost
Xception	0.938	0.925	0.918	0.92	$\tilde{2}$ h	$\tilde{2}2$ ms per image	Optimized for image processing	Requires extensive data augmentation
Inception-ResNet-V2	0.95	0.935	0.925	0.932	$\tilde{3}.5$ h	$\tilde{2}8$ ms per image	High accuracy, robust for complex tasks	High computational complexity
AlexNet	0.89	0.875	0.87	0.872	Low (2–3 h on GPU)	Very Low (40 ms)	Simple, fast training, good baseline	Limited depth, lower accuracy
VGG-16	0.91	0.9	0.898	0.902	High (12–15 h on GPU)	Moderate (120 ms)	High accuracy, well-known baseline	Large model size, high computation
ResNet-50	0.925	0.915	0.913	0.917	Moderate (6–8 h on GPU)	Moderate (100 ms)	Good balance of depth and accuracy	Requires more memory and tuning

## Conclusion

Skin cancer is the widest-spread disease all over the world. Various approaches were introduced for detecting this cancerous disease but they were not effective in appropriate identification. Hence, this paper introduced a new model, known as PCSN-Net for detecting skin cancer. Initially, the input skin cancer image is pre-processed by employing Medav filter to eradicate the noise in image. Next, cancer region is segmented by utilizing DeepSegNet, which is formed by integrating SegNet and Deep joint segmentation, where RV coefficient is used to fuse the outputs. Following this, the segmented image is augmented by including the process, namely geometric transformation, colorspace transformation, mixup and CutMix. Then the features, such as textural, statistical, LNDP, DWT based LDP with entropy are mined. At last, skin cancer detection is performed by employing PCSN-Net, which is formed by fusing PCNN and DSNN. The experimental results of PCSN-Net in terms of FPR, PPV, NPV, specificity, sensitivity, and accuracy are 0.107, 0.908, 0.897, 0.926, 0.947, and 0.957, respectively. Future work aims to develop feature selection approaches, and Artificial Intelligence (AI) based techniques can also be implemented for speeding up the cancer treatment and diagnosis process.

## Data Availability

The data will be provided by the corresponding author, Dr. Vanmathi C (email: vanmathi.c@vit.ac.in), upon reasonable request.
